# Isothermal equation of state and high-pressure phase transitions of synthetic meridianiite (MgSO_4_·11D_2_O) determined by neutron powder diffraction and quasielastic neutron spectroscopy

**DOI:** 10.1107/S2052520616018254

**Published:** 2017-01-31

**Authors:** A. Dominic Fortes, Felix Fernandez-Alonso, Matthew Tucker, Ian G. Wood

**Affiliations:** a ISIS Facility, STFC Rutherford Appleton Laboratory, Harwell Science and Innovation Campus, Chilton, Oxfordshire OX11 0QX, England; b Department of Earth Sciences, University College London, Gower Street, London WC1E 6BT, England; c Department of Physics and Astronomy, University College London, Gower Street, London WC1E 6BT, England; d Spallation Neutron Source, 8600 Spallation Dr, Oak Ridge, TN 37830, USA

**Keywords:** meridianiite, undecahydrate, enneahydrate, equation of state, neutron diffraction, quasielastic neutron scattering

## Abstract

We have collected neutron powder diffraction data from MgSO_4_·11D_2_O (the deuterated analogue of meridianiite), a highly hydrated sulfate salt that is thought to be a candidate rock-forming mineral in some icy satellites of the outer solar system. Our measurements, made using the PEARL/HiPr and OSIRIS instruments at the ISIS neutron spallation source, covered the range 0.1 < *P* < 800 MPa and 150 < *T* < 280 K. The refined unit-cell volumes as a function of *P* and *T* are parameterized in the form of a Murnaghan integrated linear equation of state having a zero-pressure volume *V*_0_ = 706.23 (8) Å^3^, zero-pressure bulk modulus *K*_0_ = 19.9 (4) GPa and its first pressure derivative, *K*′ = 9 (1). The structure’s compressibility is highly anisotropic, as expected, with the three principal directions of the unit-strain tensor having compressibilities of 9.6 × 10^−3^, 3.4 × 10^−2^ and 3.4 × 10^−3^ GPa^−1^, the most compressible direction being perpendicular to the long axis of a discrete hexadecameric water cluster, (D_2_O)_16_. At high pressure we observed two different phase transitions. First, warming of MgSO_4_·11D_2_O at 545 MPa resulted in a change in the diffraction pattern at 275 K consistent with partial (peritectic) melting; quasielastic neutron spectra collected simultaneously evince the onset of the reorientational motion of D_2_O molecules with characteristic time-scales of 20–30 ps, longer than those found in bulk liquid water at the same temperature and commensurate with the lifetime of solvent-separated ion pairs in aqueous MgSO_4_. Second, at ∼ 0.9 GPa, 240 K, MgSO_4_·11D_2_O decomposed into high-pressure water ice phase VI and MgSO_4_·9D_2_O, a recently discovered phase that has hitherto only been formed at ambient pressure by quenching small droplets of MgSO_4_(aq) in liquid nitrogen. The fate of the high-pressure enneahydrate on further compression and warming is not clear from the neutron diffraction data, but its occurrence indicates that it may also be a rock-forming mineral in the deep mantles of large icy satellites.

## Abstract 2

We report high-pressure neutron powder diffraction measurements of the most hydrated phase in the MgSO_4_–H_2_O system, magnesium sulfate undecahydrate, including an analysis of the elastic strain tensor and observations concerning phase changes that occur at high pressure.

## 1. Introduction   

### 1.1. Background   

**Figure 1:**
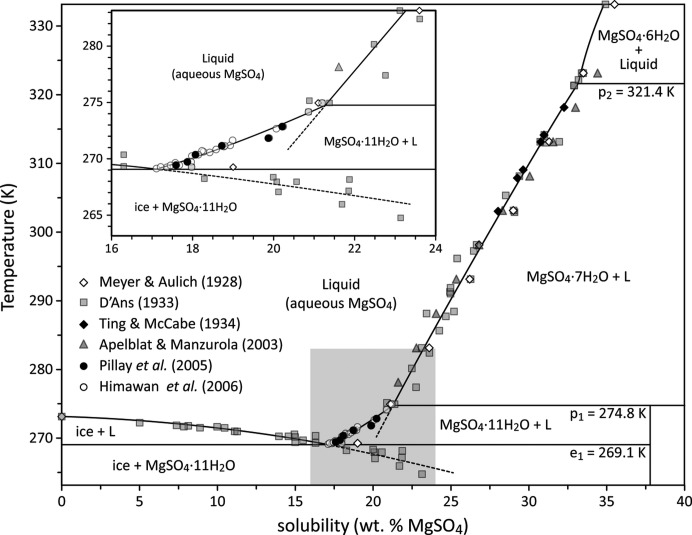
Phase diagram of the binary system MgSO_4_–H_2_O at 1 MPa. Symbols report observed equilibria and the solid lines show stable liquidi and solidi; dashed lines indicate metastable extensions of various melting curves. The composition of the liquid at the eutectic, *e*_1_, is 17.1 wt % MgSO_4_, and at peritectic *p*_1_ = 21.3 wt % MgSO_4_. ▸ ▸ ▸ ▸

Magnesium sulfate undecahydrate, MgSO_4_·11H_2_O, is the stable phase in contact with an aqueous solution of MgSO_4_ at room pressure over a narrow temperature range, between a eutectic with ice Ih at 269.1 K and an incongruent melting point at 274.8 K (Pillay *et al.*, 2005 ▸; Himawan *et al.*, 2006 ▸), as shown in Fig. 1 ▸. Meridianiite is triclinic, space group 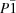 (*Z* = 2) with *a* = 6.75081 (3), *b* = 6.81463 (3), *c* = 17.29241 (6) Å, α = 88.1183 (3), β = 89.4808 (3), γ = 62.6891 (3)° and *V* = 706.450 (3) Å^3^ at 250 K (Fortes *et al.*, 2008 ▸).

This substance occurs naturally on Earth as the mineral meridianiite, being found in a variety of glacial and periglacial environments (Sakurai *et al.*, 2009 ▸; Genceli *et al.*, 2009 ▸) and in a limited number of MgSO_4_-rich hypersaline lakes during the winter months; example localities where the mineral has been identified include the Basque Lakes, Clinton Lake and kłlil’x^w^ (aka Spotted Lake), all in British Columbia, Canada (*e.g.* Peterson *et al.*, 2007 ▸; Cannon, 2012 ▸). Whilst MgSO_4_-rich saline waters are comparatively rare on Earth due to the influence of continental weathering, such liquids are expected to be common on other rocky planets where the weathering of basaltic materials dominates (King *et al.*, 2004 ▸). On Mars, abundant magnesium(II) and iron(III) sulfates are known to occur, including minerals such as kieserite and jarosite (*e.g.* Clark *et al.*, 1976 ▸; Toulmin *et al.*, 1977 ▸; Wänke *et al.*, 2001 ▸; Foley *et al.*, 2003 ▸; McSween, 2004 ▸; Chipera & Vaniman, 2007 ▸) and it is hypothesized that meridianiite may occur in a permafrost-like deposit, forming a substantial reservoir of bound water in the near-surface regolith (Feldman, Mellon *et al.*, 2004 ▸; Feldman, Prettyman *et al.*, 2004 ▸; Peterson & Wang, 2006 ▸). Similarly, water–rock interactions during the accretion and differentiation of icy planetary bodies in the outer solar system may have resulted in large brine reservoirs crystallizing substantial quantities of MgSO_4_ and Na_2_SO_4_ hydrates (Kargel, 1991 ▸). These are apparent in near-IR spectra of their surfaces (Orlando *et al.*, 2005 ▸; Dalton *et al.*, 2005 ▸; Dalton, 2007 ▸; Shirley *et al.*, 2010 ▸), although it remains unclear the extent to which some of the hydrated salts on Europa’s surface are due to endogenic *versus* exogenic processes, such as radiolysis of MgCl_2_ combined with sulfur implantation from neighbouring Io (Brown & Hand, 2013 ▸). Nevertheless, meridianiite may be a major rock-forming mineral in the mantles of many icy satellites.

As a result of this interest in MgSO_4_–brines on planetary surfaces and in planetary interiors, work has been done on the properties of liquids in solids in the MgSO_4_–water system under non-ambient conditions over the last decade. These include studies of the phase behaviour and liquid properties, principally *on* the liquidus, at pressures as high as 5 GPa and temperatures up to 600 K (Hogenboom, 1995 ▸; Dougherty *et al.*, 2007 ▸; Nakamura & Ohtani, 2011 ▸; Vance & Brown, 2013 ▸; Vance *et al.*, 2014 ▸) and including the presence of clathrate-forming volatiles (Muñoz-Iglesias *et al.*, 2014 ▸). Our group has worked on the properties of the solid phases co-existing with these potential ocean-forming brines, determining the thermal expansion and incompressibility and polymorphic phase behaviour of MgSO_4_·7H_2_O (Fortes *et al.*, 2006 ▸; Gromnitskaya *et al.*, 2013 ▸) and Na_2_SO_4_·10H_2_O (Brand *et al.*, 2010 ▸; Fortes, Brand *et al.*, 2013 ▸). Accurate knowledge of the properties of both solid and liquid phases is fundamental to computing, for example, the buoyancy of ‘igneous’ melts and the partial freezing behaviour of global subsurface oceans in the interiors of icy planetary bodies. Furthermore, the density of the solid is required in order to calculate accurately the radial density structure of a model icy satellite.

To date, however, there remains a gap in our knowledge of meridianiite’s properties at high pressure, which needs to be closed in order to address problems in planetary modelling, specifically its equation of state and phase behaviour. As part of a programme to study the high-pressure behaviour of candidate ‘planetary’ ices and hydrates, we have measured the thermal expansion of meridianiite from 4 to 250 K (Fortes *et al.*, 2008 ▸), carried out a single-crystal structural study (Fortes, Wood & Gutmann, 2013 ▸), and used density functional theory (DFT) calculations to simulate the material at high pressures (Brand, 2009 ▸). The computational study produced the first quantification of the bulk elastic properties and of the highly anisotropic compressional behaviour: a more comprehensive analysis of these calculations and comparison with this experimental work will be presented elsewhere.

This contribution is the first of three closely related works on *M* ^2+^SO_4_ cryohydrates. The second paper (Fortes *et al.*, 2017*a* ▸) deals in detail with the structure, thermoelastic properties and possible natural occurrence of MgSO_4_·9H_2_O, which is metastable with respect to MgSO_4_·11H_2_O at ambient pressure but which, as we show in this work, forms reproducibly by the decomposition of meridianiite at high pressure. The third paper (Fortes *et al.*, 2017*b* ▸) describes the Ni^2+^ analogue of MgSO_4_·9H_2_O and the solid solution series between Mg and Ni-bearing end-members, as well as the structure of NiSO_4_·8H_2_O.

### 1.2. Experimental objectives   

The objectives of this work are twofold: (i) to determine the unit-cell parameters of meridianiite as a function of pressure in order to fit an isothermal equation of state (EOS) for comparison with *ab initio* calculations; (ii) to characterize any phase transitions that occur in the pressure range of relevance to the interiors of large icy satellites, roughly 0–2 GPa. The most effective method of achieving these goals is to use neutron powder diffraction with a sample contained in a gas-pressure vessel (for *P* < 550 MPa) or in an opposed-anvil press (for *P* > 550 MPa). A necessary consequence of using neutron scattering to obtain data from a powder specimen in a complex *P*,*T* sample environment is the requirement to use a deuterated analogue, MgSO_4_·11D_2_O, in order to eliminate the undesirable incoherent neutron scattering from ^1^H. Although there are precedents where materials exhibit substantially different phase behaviour on deuteration, our experience of working with many different forms of ice and salt hydrates is that deuteration has a negligible effect on the molar volume and shifts the values of thermoelastic parameters and locations of phase boundaries by just a few percent (*e.g.* Pistorius, 1968 ▸; Fortes, Wood, Tucker *et al.*, 2012 ▸).

As in our previous study of mirabilite, we chose to make the gas-cell measurements using the OSIRIS instrument at the ISIS neutron spallation source, Rutherford Appleton Laboratory, UK (Telling & Andersen, 2005 ▸, 2008 ▸; Demmel *et al.*, 2015 ▸; Telling *et al.*, 2016 ▸). OSIRIS is both a powder diffractometer, with a high-resolution backscattering detector bank, and an inelastic spectrometer (http://www.isis.stfc.ac.uk/instruments/osiris); the instrument views a 20 K liquid-hydrogen moderator and thus receives a high flux of colder (longer wavelength) neutrons, making it well suited to the study of low-symmetry materials with large unit cells. In addition to the OSIRIS diffraction data, we obtained simultaneous quasielastic neutron scattering (QENS) data that proved to be useful in understanding the transformation that occurred on warming meridianiite on the 545 MPa isobar. QENS data are measured in an inverted backscattering geometry from a highly oriented pyrolytic graphite (HOPG) crystal analyser; specifically, the (002) reflection provides an energy resolution of 24.5 µeV in the range 0.3 < *Q* < 1.8 Å^−1^. Following subtraction of the substantial *Q*-dependent background from the TiZr pressure vessel, reduction and correction of the QENS data were done using the MODES software package (Howells *et al.*, 2010 ▸).

For the higher pressure neutron powder diffraction measurements we used PEARL/HiPr, also at the ISIS neutron source (Bull *et al.*, 2016 ▸), which is optimized for measurements using bulky high-pressure sample environments.

## 2. Experimental method   

Three high-pressure experiments were carried out using several independently prepared specimens of deuterated meridianiite. The first two of these involved measurements up to ∼ 1 GPa in a Paris–Edinburgh (P–E) opposed-anvil press (Besson *et al.*, 1992 ▸) on PEARL/HiPr, and the last experiment involved measurements up to 550 MPa in a gas pressure vessel using OSIRIS. Respectively, these are referred to as Experiment 1, 2 and 3, as detailed below.

### 2.1. Experiment 1 (PEARL/HiPr)   

Meridianiite was formed by immersion in liquid nitrogen of a polyethylene capsule containing a nearly stoichiometric solution of MgSO_4_ in D_2_O: since there is always a slight excess of water we inevitably observe crystallization of a small quantity of ice. A cylindrical block of fine-grained polycrystalline material formed in this manner was broken up with a hammer and subsequently ground to a powder under liquid nitrogen in a refrigerated workshop (*T* = 258 K) in the UCL Earth Sciences cold rooms. The powder specimen was packed into a pair of TiZr encapsulated gaskets (Marshall & Francis, 2002 ▸), pre-chilled in a 253 K chest freezer, along with a ∼ 50 mg ball of compacted Pb foil intended to act as an internal pressure standard (Fortes *et al.*, 2007 ▸; Fortes, Wood, Alfredsson *et al.*, 2012 ▸ – see also the supporting information). Due to uncertainty regarding the behaviour of the specimen in a standard 4:1 MeOD/EtOD pressure medium, the loading was left ‘dry’, which subsequently resulted in poor pressure generation under loads smaller than ∼ 20 tonnes. The filled gaskets were mounted between two WC anvils and bolted together between steel plates. This clamped assembly was transported to the ISIS facility immersed in liquid nitrogen.

At ISIS, the pre-assembled loading jig, at ∼ 100 K, was transferred quickly into the load frame of a V4 P–E press and a sealing load of 6 tonnes was applied. The press was then lowered into a cryogenic tank until partially immersed in liquid nitrogen.

Diffraction patterns obtained at 177 K under a load of 7 tonnes exhibited strong Bragg peaks with widths close to the instrumental resolution, suitable for unit-cell refinement. Increasing the load at 200 K resulted in considerable line broadening, and so the specimen was warmed to 240 K, where the Bragg peaks became sharp once more. Under a load of 31 tonnes (∼ 1.0 GPa from the Pb pressure marker), the diffraction pattern of meridianiite changed substantially from that observed at 27 tonnes.

### 2.2. Experiment 2 (PEARL/HiPr)   

A specimen of meridianiite was formed and loaded in essentially the same manner as for Experiment 1, the principal difference being that we used 3M^TM^ Fluorinert^TM^ liquid FC-87 as a pressure-transmitting medium instead of leaving the sample ‘dry’. The cell was sealed under a load of 6 tonnes and equilibrated at a temperature of 240 K. Inspection of the diffraction pattern revealed strong Bragg peaks from MgSO_4_·11D_2_O and data were collected at this *P*,*T* point for 17 h (2722 µA h) in order to provide a high-quality reference pattern. Later refinement revealed the presence of accessory ice V in the specimen, which is consistent (at this temperature) with the pressure of 560 MPa obtained from the Pb pellet. The load was then increased first to 8 tonnes and later to 10 tonnes at 240 K. Due to the tight packing of the gaskets, and the use of Fluorinert, pressure was taken up immediately; the phase transition observed in Experiment 1 occurred at 10 tonnes (∼ 900 MPa sample pressure) and data were collected at this *P*,*T* point for 12 h (1921 μA h). Subsequent warming to 260 and 280 K at a load of 10 tonnes resulted in no further change in the diffraction pattern. Another 12 h integration was acquired at 10 tonnes, 280 K. Finally, the specimen was compressed, at 280 K, under loads of 15, 20 and 25 tonnes, with no significant change in the diffraction pattern being observed.

### 2.3. Experiment 3 (OSIRIS)   

Single crystals of deuterated meridianiite were grown from solutions of MgSO_4_ (Sigma-Aldrich M7506, anhydrous β-phase, *ReagentPlus* ^®^, ≥ 99.5%) in D_2_O (Aldrich 151882 99.9 atom % D) at temperatures of 272 K in the UCL Earth Sciences cold rooms. The solutions were sealed in plastic bags with a desiccant (MgCl_2_ powder) and allowed to evaporate until crystals of MgSO_4_·11H_2_O appeared. These crystals were harvested and dried on filter paper before being moved to a refrigerated chamber maintained at 258 K. The dried crystals were immersed in liquid nitrogen in a steel cryomortar and ground to a powder with a nitrogen-cooled steel pestle.

Powdered MgSO_4_·11D_2_O was transferred into a TiZr null-scattering alloy gas-pressure vessel embedded in dry-ice snow. The pressure vessel was kept at dry-ice temperatures whilst it was screwed onto a cryostat centre stick and fitted with copper collars (top and bottom), RhFe resistance thermometers and heaters to allow temperature control. This assembly was then inserted into a standard Orange cryostat of 100 mm bore, mounted on the OSIRIS beamline, and equilibrated at 240 K under 48 MPa of He gas. Gas pressures were measured with a transducer on the top of the gas-handling cryostat centre stick, which has an error of 0.3%.

First inspection of the powder diffraction data revealed that the specimen consisted primarily of meridianiite with a small admixture of ice Ih. Beginning at 48 MPa, data were collected in roughly 50 MPa increments along the 240 K isotherm up to a maximum pressure of 550 MPa, counting each datum in two separate time-of-flight bands (known as *d*-range 2 and *d*-range 3); counting times were ∼ 2 h in each *d*-range, equivalent to 150 µA of integrated proton current at the instrument’s 25 Hz operating chopper frequency in each time window. The time-of-flight bandwidth of *d*-range 2 is 29.4 to 69.4 ms, corresponding to *d*-spacings of 1.8 to 4.0 Å in the backscattering detectors (150 < 2θ < 171°), and the time-of-flight bandwidth of *d*-range 3 is 47.1–87.1 ms, corresponding to *d*-spacings of 2.9–4.9 Å.

At 550 MPa, the temperature was then increased from 240 to 275 K in 5 K increments counting each for 1 h in *d*-range 2 only, and subsequently the temperature was raised to 295 K. Since there was a substantial change in the diffraction pattern at 270 K, which appeared to involve partial melting of the specimen, we collected data at 295 K for ∼ 3.5 h in *d*-range 2 followed by 2 h in *d*-range 3.

The powder diffraction data were normalized to the upstream monitor, corrected for instrumental efficiency using data from a vanadium standard, and finally corrected for the substantial wavelength-dependent absorption of the TiZr vessel by subtraction of a histogram collected from the empty pressure cell at room temperature.

### 2.4. Refinement   

**Figure 2:**
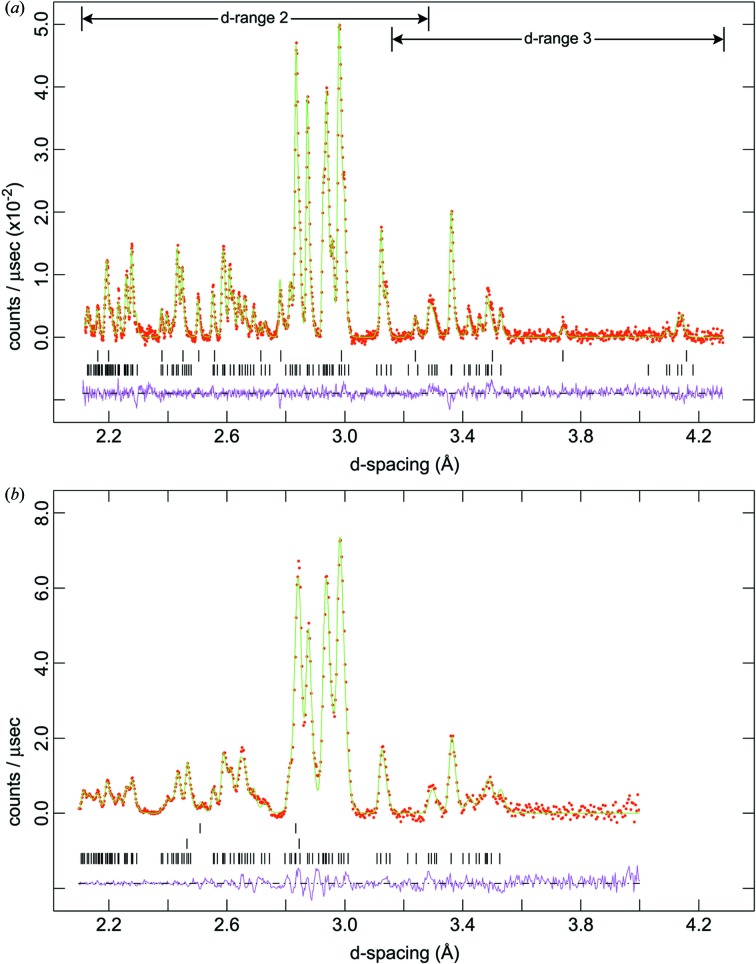
Comparison of neutron powder diffraction data obtained on (*a*) OSIRIS and (*b*) PEARL/HiPr at approximately the same pressure and temperature (∼ 0.5 GPa, 240 K). Red points are the measured data, green lines are the model fits and the pink lines underneath the data are the model residuals. Tick marks in (*a*) indicate the expected positions of Bragg reflections from meridianiite (bottom) and metastable ice Ih (top). Tick marks in (*b*) indicate Bragg reflections from meridianiite (bottom), the Pb pressure marker (middle) and tungsten carbide from the anvils (top).

Data were exported in a format suitable for analysis with the *GSAS*/*Expgui* software package (Larsen & Von Dreele, 2000 ▸: Toby, 2001 ▸). A comparison of the neutron powder diffraction data from OSIRIS and PEARL/HiPr is shown in Fig. 2 ▸.

The diffraction data were fitted initially using the Rietveld method (Rietveld, 1969 ▸), to obtain accurate peak shifts from one *P* or *T* increment to the next, and the least-squares minimization was then run to convergence using the ‘*F*(calc)-weighted’ profile refinement method, varying only the peak-profile coefficients, and unit-cell parameters of the phases present (meridianiite and one of several ice phases).

In order to ensure accurate lattice parameters for meridianiite in each experiment and to ensure accurate pressures derived from the Pb lattice constant, it was necessary to refine the diffractometer constants for each sample loading. Since it is never possible in practice to mount the high-pressure apparatus – and hence the samples – in exactly the same position, there are minute variations in the neutron flight path from one loading to the next. Additionally, the bulk composition and packing density of samples is never identical, resulting in small variations in specimen absorption; this results in shorter wavelength neutrons penetrating further into the sample, and ultimately traversing a longer flightpath than long-wavelength neutrons. The refineable parameters DIFC and DIFA correct for these variations provided that we have a set of reference lattice parameters for either the sample and/or an internal standard measured under comparable conditions. Since the thermal expansion of Pb is well known, and we have highly accurate and precise lattice parameters for meridianiite, measured as a function of temperature at room pressure (Fortes *et al.*, 2008 ▸), we are able to use the zero-P reference measurements from each high-pressure experiment to obtain DIFC and DIFA.

## 3. Results   

### 3.1. Isothermal equation of state and elastic strain figure   

**Figure 3:**
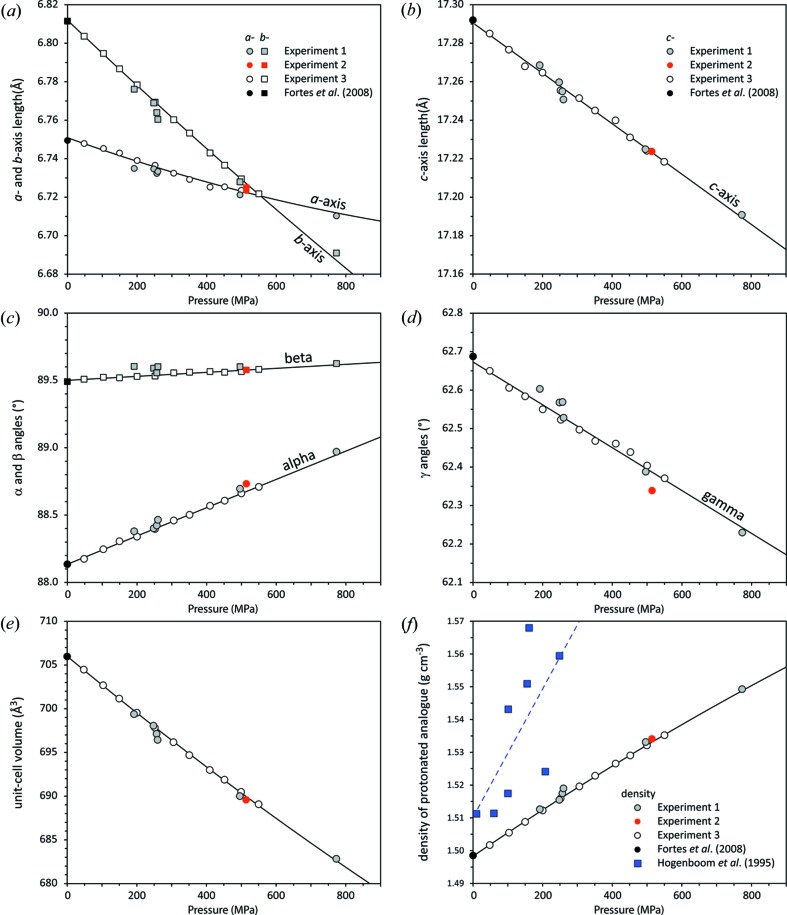
Plot of unit-cell parameters of MgSO_4_·11D_2_O, the filled and open symbols corresponding to the data listed in Table S1. Solid lines are polynomial fits to the data (coefficients in Table 1 ▸).

Experiments 1 and 2 yielded unit-cell parameters for deuterated meridianiite at seven state points between 200 and 800 MPa at 240 K: the data collected in Experiment 3 provided unit-cell parameters at 11 state points between 50 and 550 MPa at 240 K (Table S1). These data are reported graphically in Fig. 3 ▸.

An initial impression of the material’s stiffness may be obtained by parameterizing the pressure-dependence of the unit-cell volume using a Murnaghan integrated linear equation of state, MILEOS (Murnaghan, 1944 ▸)
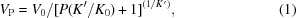
where *K*_0_ is the zero-pressure isothermal bulk modulus, and *K*′ is the first pressure derivative of the bulk modulus, (∂*K*_0_/∂*P*). A fit of equation (1), using *OriginPro* (OriginLab, Northampton, MA), to the experimental *V*(*P*) obtained solely from Experiment 3 yields *V*_0_ = 706.23 (8) Å^3^, *K*_0_ = 19.9 (4) GPa and *K*′ = 9 (1) at 240 K. This is somewhat more compressible than synthetic epsomite, MgSO_4_·7D_2_O (*K*_0_ = 22.6 GPa and *K*′ = 5.3), and marginally stiffer than synthetic mirabilite, Na_2_SO_4_·10D_2_O (*K*_0_ = 19.1 GPa and *K*′ = 5.8), both sets of values also at 240 K. Fig. 3 ▸(*f*) shows the variation in density of protonated meridianiite as a function of pressure calculated from our observations of MgSO_4_·11D_2_O assuming there is no difference in molar volume between the deuterated and protonated isotopologues. For simple calculations, the density can be described by a polynomial where ρ(*P*) = 1.4983 (1) + 7.2 (1) × 10^−1^ *P* − 9 (2) × 10^−3^ *P* ^2^ (g cm^−3^), where *P* is in GPa. Shown for comparison in Fig. 3 ▸(*f*) are the density values obtained by Hogenboom (1995 ▸); evidently the pressure-dependence reported in that work is quite inaccurate, their linear fit through the highly scattered points corresponding to a bulk modulus of ∼ 8 GPa.

For a crystal of triclinic symmetry the three principal elastic strain moduli are free, whilst remaining orthogonal to one another, to adopt any orientation with respect to the crystallographic reference frame; thus an accurate picture of the structure’s compressional behaviour is not readily obtained simply by fitting equations of state to the individual axial lengths as a function of pressure, as we have done with the volume. Instead, we must determine the shape and orientation of the compressibility tensor’s representation surface (hereafter, in the interest of brevity, described as the ‘unit-strain figure’), which for a triclinic crystal is described by a second rank tensor of the form
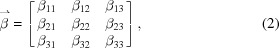
in which the Eulerian infinitesimal unit-strains, or compressibility coefficients, β_12_ = β_21_, β_13_ = β_31_ and β_23_ = β_32_. The eigenvalues and eigenvectors of this matrix, obtained by matrix decomposition methods, are the magnitudes and orientations of the three principal axes of the unit-strain figure (*i.e.* directional compressibilities, 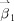, 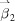 and 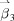) with respect to an orthogonal basis defined according to the Institute of Radio Engineers’ convention, which orients *X* || a* and *Z* || *c*. More specifically, we have applied the commonly used transformation matrix, **A**, relating the orthogonal basis and the unit cell of a triclinic crystal given by Boisen & Gibbs (1990 ▸)
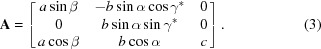
Each of the unit-cell parameters in Table S1 was fitted either with a quadratic polynomial expression of the form 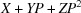 (*a*-axis, *b*-axis) or a linear expression of the form 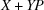 (*c*-axis, α, β, γ); the parameters resulting from these fits are listed in Table 1 ▸. The solid lines in Figs. 3 ▸(*a*)–(*d*) represent these fits and the solid line in Fig. 3 ▸(*e*) represents the unit-cell volume computed from the fits.

The fit parameters in Table 1 ▸ were used to calculate smoothly varying unit-cell parameters as a function of pressure, from which the coefficients in equation (2) were obtained using the methods described by Schlenker *et al.* (1978 ▸) and Hazen & Finger (1982 ▸); the magnitudes of the principal compressibilities, 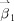, 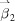 and 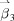, and their spatial orientation with respect to the orthogonal basis were then obtained by standard eigenvalue decomposition methods.

**Figure 4:**
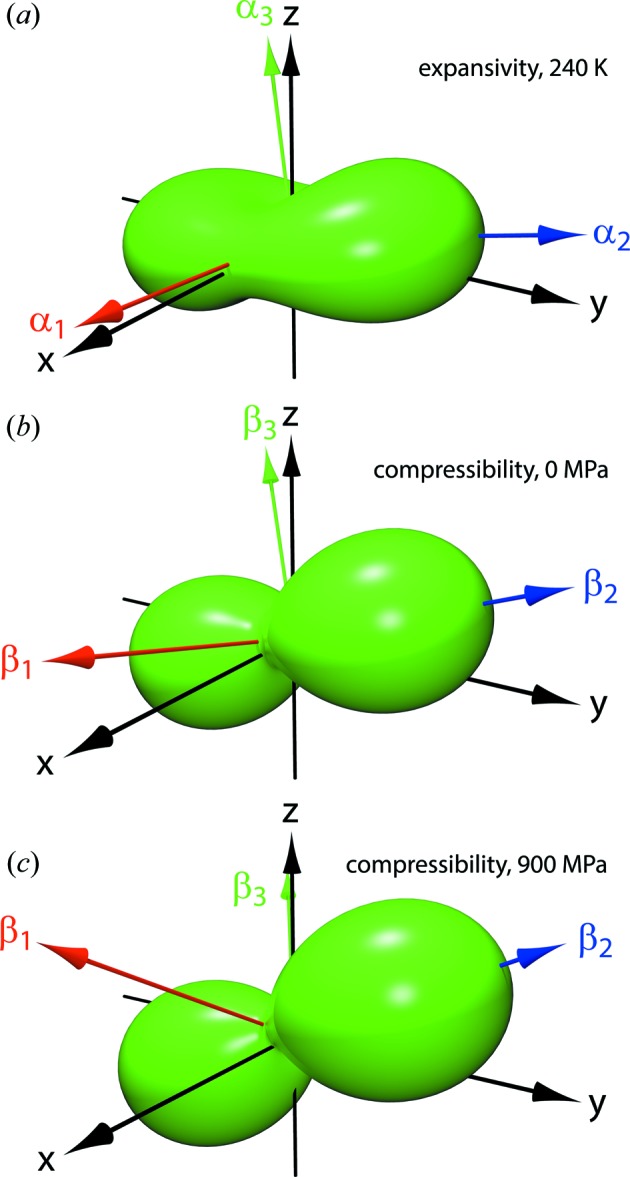
Representation glyphs of the unit-strain tensor surface corresponding to (*a*) thermal expansion at 240 K (after Fortes *et al.*, 2008 ▸), (*b*) compressibility at zero pressure and (*c*) compressibility at 900 MPa. Black arrows indicate the orthogonal reference frame [*cf.* Equation (3) and Fig. 5 ▸ *a*]. Coloured arrows show the eigenvectors of the Eulerian infinitesimal unit-strain tensor. Representation surfaces created using *WinTensor* (Kaminski, 2004 ▸).

The principal and volumetric compressibilities and incompressibilities (*e.g.* 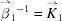) were fitted with linear functions in *P* to obtain the zero-pressure parameters and their first derivatives (Table 2 ▸). It is worth observing that this method recovers reasonably accurately the value of *K*_0_ found earlier by fitting the Murnaghan integrated linear EoS. Representation surfaces (Reynolds glyphs – see Hashash *et al.*, 2003 ▸) of the unit-strain figures at zero pressure and at 900 MPa are shown in Fig. 4 ▸ along with a similar representation of the unit-strain due to a change in temperature (*i.e.* the thermal expansion; Fortes *et al.*, 2008 ▸), where the distance from the origin to the edge of the representation surface indicates the compressibility (or thermal expansion) in any given direction; the disposition of the orthogonal reference frame and the principal axes are indicated by labelled arrows.

**Figure 5:**
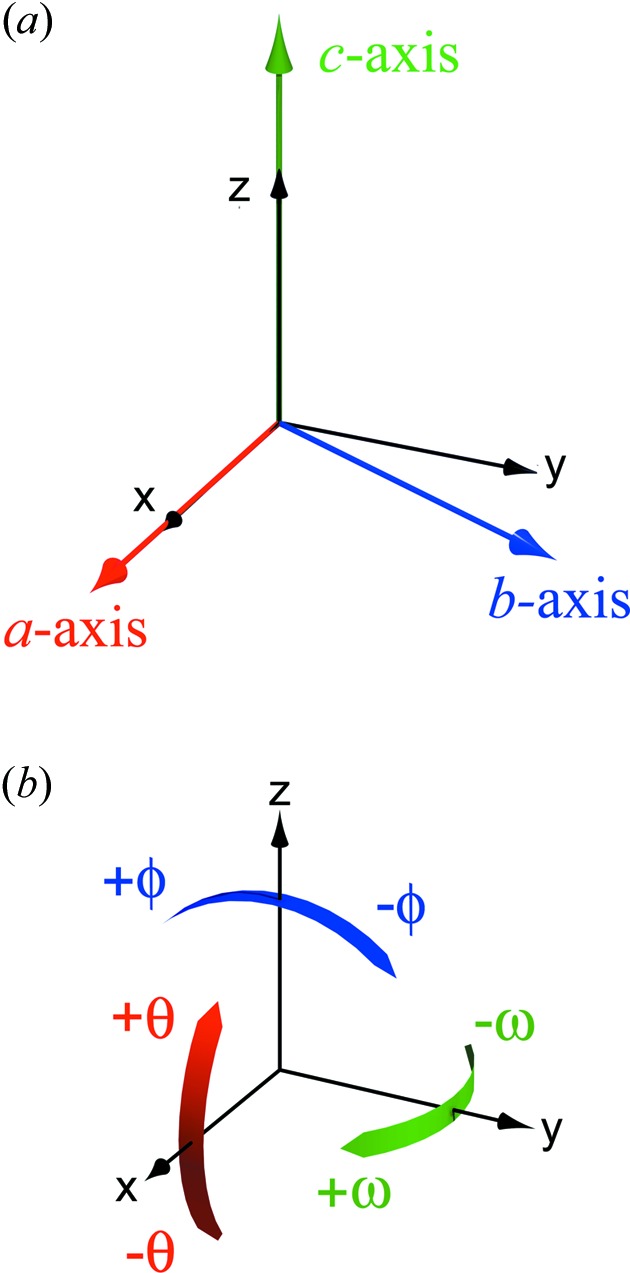
(*a*) Relationship between the crystallographic axes and the orthogonal basis used for the strain calculations. (*b*) Cartoon illustrating the notation used to describe the orientation of the compressibility tensor’s principal directions with respect to the orthogonal reference frame.

**Figure 6:**
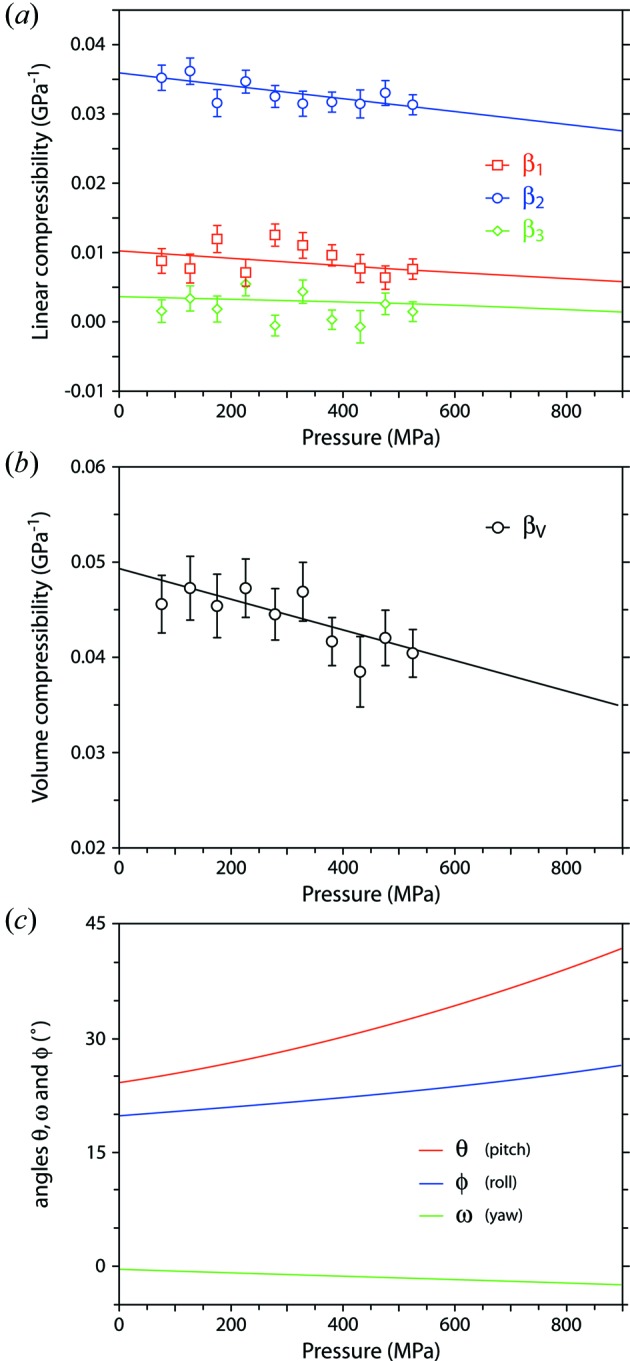
(*a*) Derived magnitudes of the compressibility tensor eigenvalues as a function of pressure, where open symbols with error bars are calculated using data in Table S1 (experiment 3) and the solid lines are calculated from the parameters in Table 2 ▸. (*b*) Volume compressibility of MgSO_4_·11D_2_O obtained as the sum 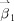 + 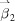 + 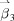. (*c*) Parameters describing the orientation the principal directions with respect to the orthogonal reference frame. Raw data are not given since these have a large scatter and large uncertainties.

The orientation of the unit-strain figure’s principal directions may be described in terms of Tait–Bryan–Cardan angles of extrinsic rotation with respect to the Cartesian reference frame referred to previously. If we adopt the aeronautical convention, such that the positive X-direction is ‘forward’, the positive Y-direction is ’left’ and the positive Z-direction is ‘up’, then these rotations may be thought of in simple descriptive terms as pitch (θ), roll (ϕ) and yaw (ω) (see Fig. 5 ▸) such that a tilt of the unit-strain figure in the positive-θ direction with changing pressure might be described as a pitch-up or nose-up *etc*. The pressure dependences of the principal and volumetric strains, and the extrinsic rotation angles, are reported in Fig. 6 ▸.

As both Figs. 4 ▸(*b*) and 6 ▸(*c*) reveal, the unit-strain figure changes its orientation with pressure, this change being characterized by: (i) a pitch-up from 14° at zero pressure to 32° at 900 MPa (mean rate = 21.1° GPa^−1^), although this is difficult to discern in Fig. 4 ▸ since the 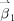 direction has quite a small compressibility; (ii) a roll to the right, increasing the bank angle slightly from 20 to 26° (mean rate = 7.3° GPa^−1^), this being particularly apparent from Fig. 4 ▸ by virtue of 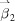 having the largest compressibility; (iii) a modest yaw to the left increasing the ‘nose-left sideslip’ from −0.4 to −2.4° (mean rate = −2.3° GPa^−1^).

### 3.2. Relationship of the elastic strain to the crystal structure   

Meridianiite consists of Mg(H_2_O)_6_^2+^ octahedra and SO_4_^2−^ tetrahedra with five additional ‘interstitial’ lattice water molecules per formula unit; such a high water to cation ratio is fairly uncommon and it is a matter of interest to examine the way in which the excess water organizes itself and the effect that this has on the material’s elastic properties. In the majority of inorganic hydrates the cation is either fully saturated (*i.e.* its first coordination shell is entirely filled by water O atoms) or is undersaturated, such that some of the cation-coordinated O atoms are O^2−^ ions rather than neutral H_2_O molecules; this tends to result in corner- and/or edge-sharing polyhedra. In ‘cation-saturated’ hydrates, the water molecules generally donate hydrogen bonds to the anions (or oxyanions) and less frequently donate hydrogen bonds to other water molecules, thus the role of water is to act as a bridge between the ions rather than associating with any neighbouring neutral water.

In hydrates where there is excess water above that required to saturate the cation, the question then arises as to whether the additional interstitial water just serves to extend these bridges or whether there is an opportunity for association with neighbouring water to form some kind of polymeric unit (*e.g.* clusters, ribbons or sheets).

**Figure 7:**
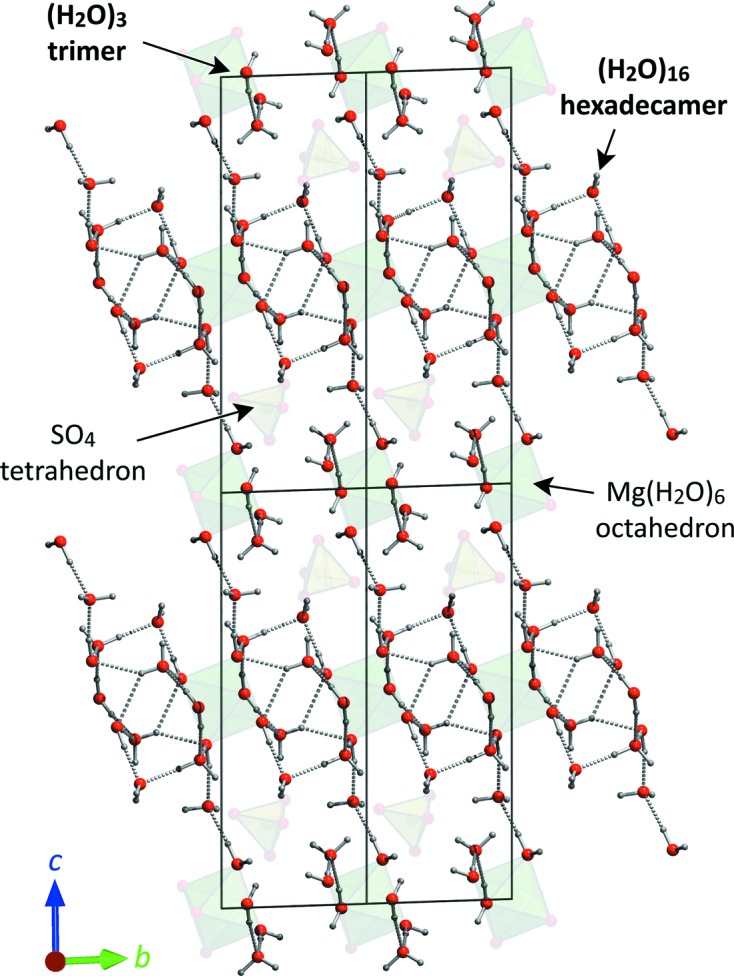
The structure of meridianiite, viewed approximately along the *a*-axis, showing four unit cells. The coordination polyhedra, Mg(H_2_O)_6_ in green and SO_4_ in yellow, are shown faint, whereas the two water clusters are emphasized. Structure from single-crystal neutron diffraction data (Fortes, Wood & Gutmann, 2013 ▸: ICSD code 236334).

In meridianiite the water molecules cluster into two types of polymeric unit, a smaller trimer and a larger centrosymmetric hexadecamer, (H_2_O)_16_. Fig. 7 ▸ highlights the geometry of these polymers and their structural relationship with the two varieties of coordination polyhedra. Since the Mg(H_2_O)_6_ octahedra have a polyhedral bulk modulus of ∼ 60 GPa and the SO_4_ tetrahedra have a polyhedral bulk modulus of ∼ 190 GPa (Brand, 2009 ▸), it is quite clear that the bulk of the crystal’s compression (recall that K_0_ ≃ 20 GPa) must be taken up by the hydrogen-bonded water framework; hence we would expect the elastic strain tensor to exhibit some spatial relationship to the large water polymer structure. Note that this differs substantially from the example of mirabilite, Na_2_SO_4_·10H_2_O, where the Na(H_2_O)_6_ coordination polyhedra are actually *more* compressible than the bulk structure (Fortes, Brand *et al.*, 2013 ▸).

**Figure 8:**
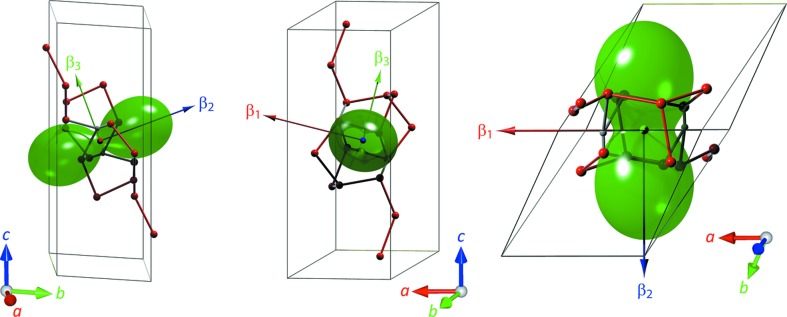
Depiction of the compressibility representation surface at 0 MPa superimposed on the unit cell of meridianiite where only the central water hexadecamer is shown. This emphasizes that the stiffest direction is along the long axis of the cluster and the most compressible direction is parallel to the two bifurcated hydrogen bonds.

In order to better appreciate the relationship between this representation surface and the crystal structure, the two are shown superimposed in Fig. 8 ▸. At zero pressure, the 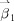
–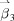 plane is almost coincident with the crystal’s (011) plane and the 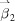
–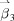 plane is within 0.25° of the (523) plane. As the illustration shows, the stiffest direction, 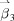, is closely aligned with the long axis of the hexadecamer whilst the most compressible direction, 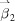, is parallel with the short axis of the hexadecamer that happens to be buttressed by two bifurcated hydrogen bonds. Thus, as expected, the magnitude and orientation of the unit-strain tensor appears to be entirely controlled by the large polymeric water cluster in the structure.

### 3.3. Comparison with DFT calculations   

These experimental results are in good qualitative agreement with the DFT calculations (Brand, 2009 ▸), inasmuch as the calculated pressure-dependences of the unit-cell parameters, the large elastic anisotropy and the orientation of the strain figures are reproduced with a degree of accuracy. For example, the DFT calculations show the *b*-axis length decreasing faster than the *a*-axis length with pressure, such that they cross over at ∼ 3 GPa (*cf.* 0.6 GPa experimentally), and α and β both increase (α faster than β) whereas γ decreases. In terms of the unit-strain figure, the shape and orientation are qualitatively very similar and the calculated unit-strain figure undergoes the same sense of ‘pitch up’ and ‘roll to the right’ as observed experimentally. Quantitatively, however, the agreement is poorer than we have found for other related hydrogen-bonded inorganic hydrates. The DFT values, for comparison with the results of this work given in Table 2 ▸, yield 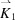 = 80 GPa, 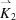 = 42 GPa, 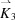 = 146 GPa and *K*_V_ = 23 GPa (with *K*′ = 4.2). In other words, the DFT calculations produce a structure that is around 10% stiffer overall with some quite substantial differences in the absolute values of the linear incompressibilities. It is worth bearing in mind that the calculations are effectively athermal (*i.e.* at 0 K), whereas the experiments are done at 240 K, a homologous temperature (*T*/*T*_melt_) = 0.886. It is perfectly reasonable to expect the compressibilities to change substantially over this temperature interval. It would be useful, in principle, to measure the elastic constants of meridianiite as a function of temperature in order to quantify this. The extent of the agreement between experiment and theory is typical of DFT calculations done using GGA functionals.

### 3.4. Phase behaviour   

**Figure 9:**
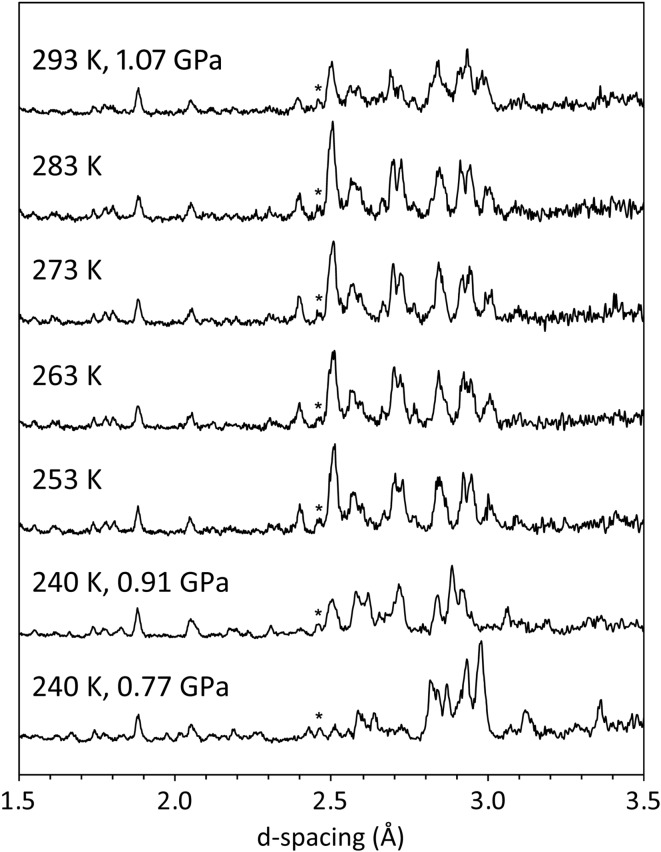
Diffraction patterns acquired in the Paris–Edinburgh press from meridianiite (the Pb pressure marker is indicated with an asterisk). The lowermost pattern shows the expected peaks from meridianiite, which transform to a new phase on increasing the pressure from 0.77 to 0.91 GPa at 240 K. Further warming (the top five data sets) show the development of a new diffraction pattern, with signs of incipient melting close to room temperature at 1.1 GPa manifested by a reduction in the intensity of all Bragg peaks, which is more substantial for the inferred contributions from ice VI.

In each of the experimental runs, meridianiite was observed to transform in some fashion that apparently involved either exsolution of high-pressure ice or else incongruent melting. The initial observation made was that compression of meridianiite along the 240 K isotherm led to a phase change at 0.85–0.95 GPa; warming of these specimens under a constant load subsequently resulted in the onset of partial melting at room temperature (Fig. 9 ▸). The initial phase change proved to be reproducible in both the dry loading and the loading where Fluorinert was used.

**Figure 10:**
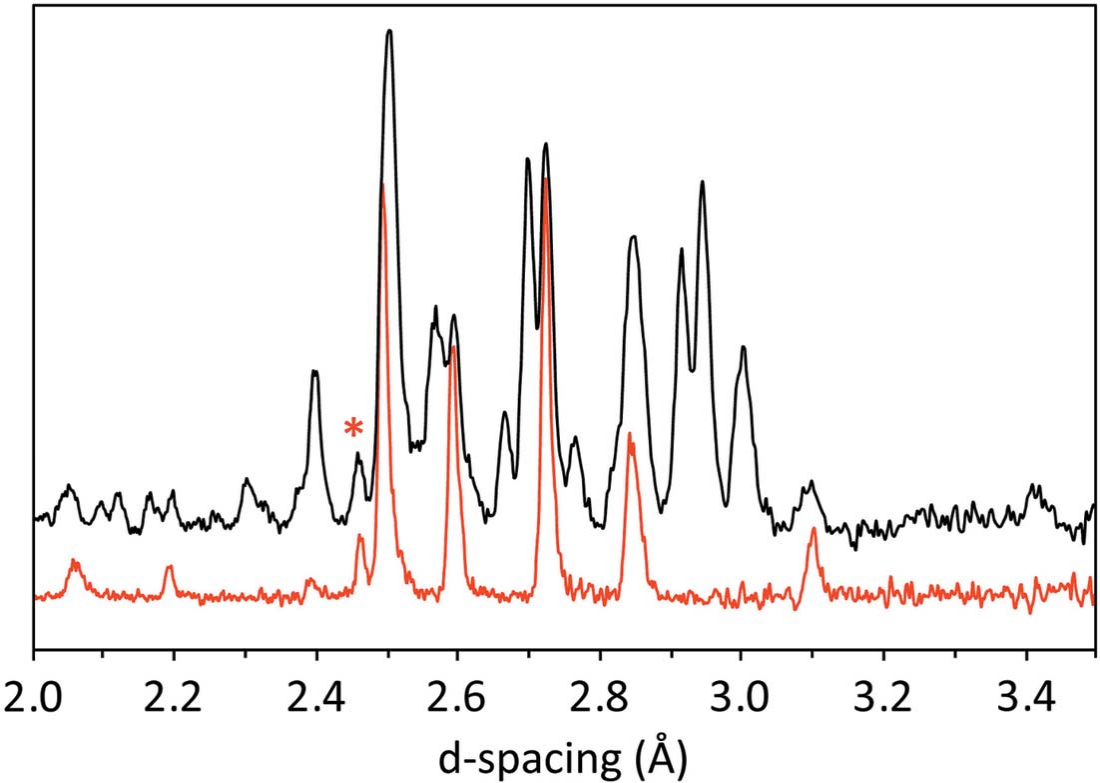
Comparison of the neutron powder diffraction pattern from the product of meridianiite’s transformation at ∼ 280 K and 1 GPa (black) and the neutron powder diffraction pattern of ice VI in red (from Fortes *et al.*, 2012 ▸) under similar *P*,*T* conditions.

The presence of Bragg peaks in the diffraction data that resemble the neutron powder pattern of ice VI (Fig. 10 ▸) led us to suspect that MgSO_4_·11D_2_O has decomposed into a mixture of D_2_O ice and some lower hydrate. There are abundant examples of water-rich materials exsolving ice at high pressure, the best known being the clathrate hydrates where the guest to water ratio increases from 1:5.75 to 1:3.5 and then 1:2 as the material expels ice in a series of high-pressure transformations (*e.g.* Loveday & Nelmes, 2008 ▸). Even amongst ‘hard’ Earth materials there are precedents for compounds to expel component oxides under pressure; the transformation from MgSiO_4_ → MgSiO_3_ + MgO at 670 km depth inside the Earth is of the most profound significance for our planet’s internal structure and convective heat transport (*e.g.* Wolstencroft & Davies, 2011 ▸).

**Figure 11:**
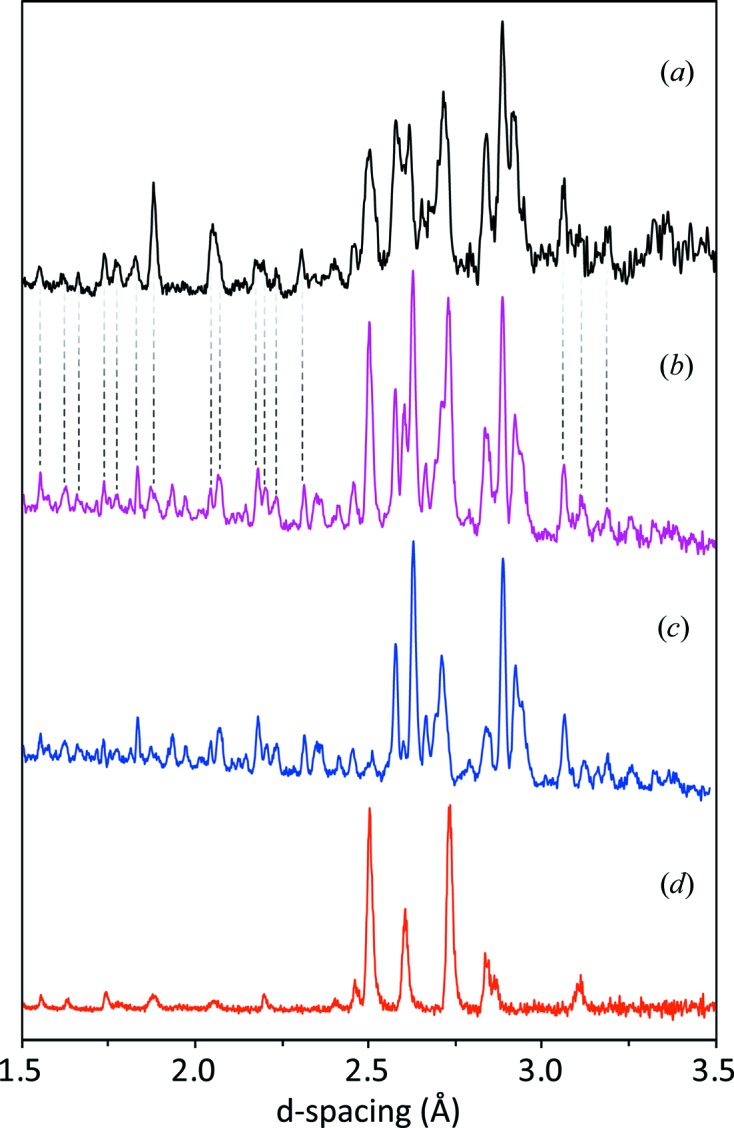
Comparison of powder diffraction data obtained in Experiment 1 (*a*), directly after the pressure-induced breakdown of meridianiite at 0.91 GPa, 240 K, and a linear sum (*b*) of the neutron powder diffraction patterns of MgSO_4_·9D_2_O (*c*), with a small shift in *d*-spacing to account for the pressure difference (see text) and D_2_O ice VI (*d*).

The initial uncertainties concerning the identity of this hydrate were resolved with our discovery of MgSO_4_·9H_2_O and measurements made on that substance at high pressure that allowed us to be certain it is the product of meridianiite’s decomposition (Fortes *et al.*, 2017*a* ▸). Shown in Fig. 11 ▸ is the 0.91 GPa, 240 K dataset from Experiment 1 (*cf*. Fig. 9 ▸, second plot from the bottom) along with the 543 MPa, 240 K dataset from MgSO_4_·9D_2_O measured in a TiZr gas cell on the HRPD beamline at ISIS (Fortes *et al.*, 2017*a* ▸). In order to obtain the closest correspondence in peak positions the latter powder pattern has been shifted by Δ*d*/*d* = −0.006, which corresponds (assuming elastic isotropy) to Δ*V*/*V* = −0.018 or, for a pressure difference of ∼ 370 MPa, a bulk modulus, *K* = *V*(Δ*P*/Δ*V*) = 20 GPa, which is a sensible value. Additional peaks in the PEARL/HiPr data are clearly from ice VI, albeit rather broad and perhaps also exhibiting preferred orientation.

Hence, the higher-pressure portion of the peritectic is described by the reaction MgSO_4_·11H_2_O → MgSO_4_·9H_2_O + ice VI. What happens when this is warmed (253–283 K in Fig. 9 ▸) is less clear. It *appears* that the enneahydrate phase transforms into something else and the peaks due to ice VI increase in intensity. This may reflect further exsolution of water from the hydrate, sharpening of the ice peaks or textured grain growth, whilst the hydrate may be a polymorph of MgSO_4_·9D_2_O, another unknown hydrate or simply the same phase exhibiting highly anisotropic thermal expansion. Further work, ideally involving high-pressure synthesis and recovery to ambient pressure at liquid-nitrogen temperatures is required. Similarly, further work to survey the high-pressure peritectic in pressure and temperature is warranted.

**Figure 12:**
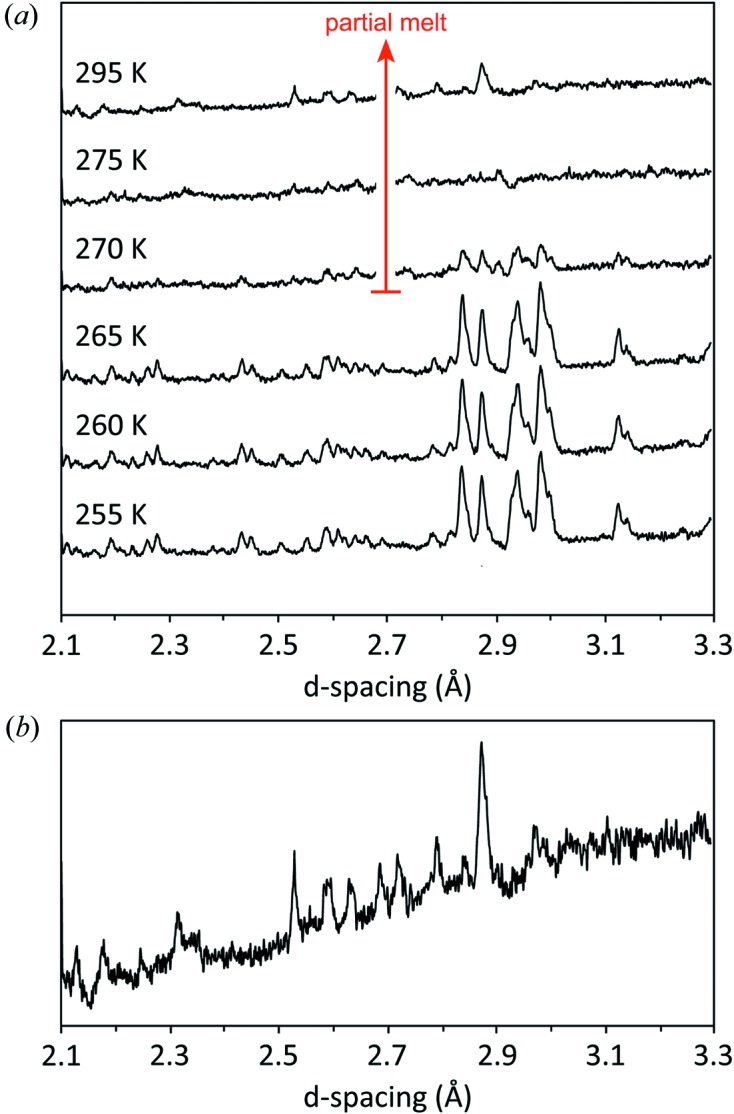
(*a*) Diffraction data obtained in the TiZr gas cell during warming of meridianiite at 545 MPa. The bottom three datasets show the expected peaks due to meridianiite; the onset of partial melting is indicated by the dramatic loss of intensity at 270 K. At 295 K only weak residual peaks remain; these are shown in an expanded view in (*b*). The identity of this phase remains unknown.

One of the goals of the gas-cell study was to survey the peritectic at lower pressures. However, warming the specimen at 545 MPa resulted in partial melting at a transition we interpret as being a peritectic. Fig. 12 ▸ shows the sequence of diffraction patterns acquired on warming from 255 to 295 K at 545 MPa; melting clearly occurs between 265 and 270 K. Residual Bragg peaks are still present at room temperature, although of much diminished intensity. Since the entire length of the sample was illuminated by the neutron beam, this indicates that the amount of residual crystalline solid is small (rather than having sunk out of view of the incident beam), which means that the solubility of this phase must be similar to the bulk composition of the specimen (95 wt % meridianiite, 5  wt % ice) at this *P*,*T* point, *i.e.* roughly 35 wt % MgSO_4_. This is substantially higher than the *ca* 27 wt % solubility of MgSO_4_ in water at room *P*,*T* or the value of *ca* 20 wt % obtained at 400 MPa by Hogenboom (1995 ▸). The implication of this is that our partial melt was highly supersaturated with respect to the heptahydrate and would have crystallized if left for a longer period.

The product of meridianiite’s partial melting at 545 MPa remains unidentified; there are few obvious similiarities with the enneahydrate diffraction patterns reported in our companion paper (Fortes *et al.*, 2017*a* ▸) or with any of the diffraction patterns shown in Figs. 9 ▸ or 10 ▸. It is worth bearing in mind that NiSO_4_·9H_2_O transforms *via* an octahydrate to the stable heptahydrate (Fortes *et al.*, 2017*b* ▸) so it is possible that we observe a similar sequence here.

**Figure 13:**
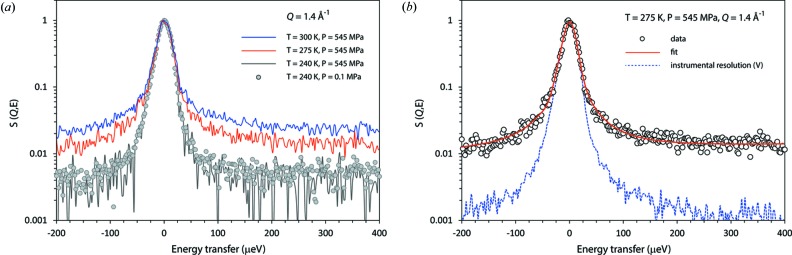
(*a*) Representative QENS datasets acquired at *Q* = 1.4 Å^−1^ as a function of *P* and *T*, revealing the broadening that occurs only when *T* ≥ 275 K at 545 MPa. (*b*) Example fit to QENS data at *Q* = 1.4 Å^−1^ at 275 K, 545 MPa, with the instrumental resolution shown for comparison as the dashed blue line.

When it became clear that there had been a dramatic change of state of the specimen, analysis of OSIRIS QENS data was used to guide our interpretation. Below the transition, at 240 K, the spectra exhibit no broadening above the instrumental resolution, indicative of negligible molecular motion on the timescales to which the measurement is sensitive (0.5–75 ps). However, above the transition, at 275 K, the QENS data exhibit a quasielastic contribution from the sample, resulting in a spectral broadening (Fig. 13 ▸).

To the best of our knowledge, this is the first time that such a signal has been observed at high pressure in conjunction with high-resolution neutron powder diffraction data.

**Figure 14:**
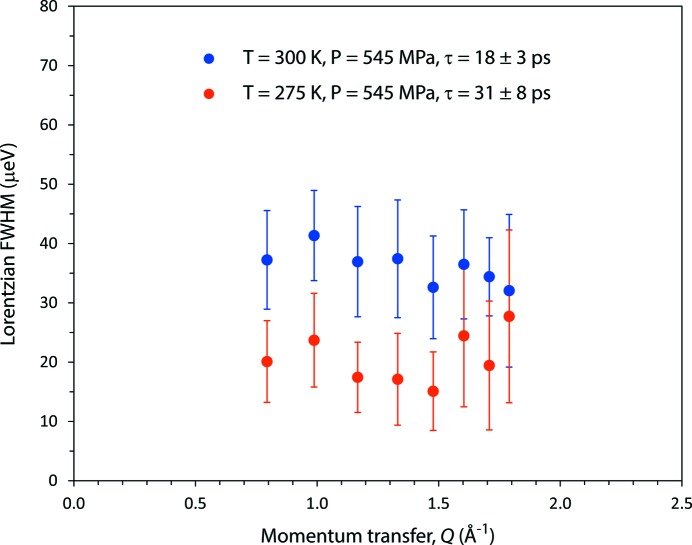
*Q*-dependence of the QENS half-widths at 275 and 300 K, 545 MPa.

The spectra continue to broaden on warming to 300 K; the most plausible explanation is the onset of re-orientational motion of D_2_O molecules above the transition temperature, the characteristic time-scales associated with the observations being τ = 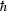/Γ_FWHM_ ≃ 32 ps at 275 K and τ ≃ 18 ps at 300 K. These time-scales are a factor of a few greater than the slowest relaxation timescales in pure liquid water (∼ 8 ps; Vinh *et al.*, 2015 ▸) at similar temperatures. Typically, the identification of melting, *i.e.* the distinction between a liquid phase and a glassy solid, rests on the detection of long-range diffusional motion, which is manifested in the presence or absence, respectively, of a *Q*-dependence to the QENS spectral widths. In a crystalline or glassy solid where atoms oscillate around their equilibrium positions in some harmonic-like potential well, there will be no dependence of the half-widths on momentum transfer. In the case of a liquid, with translational diffusion of molecular centres of mass, then a *Q*-dependence of the half-widths will be seen, scaling as *Q* ^2^ at low *Q* (see *e.g.* Fernandez-Alonso & Price, 2013 ▸; Johnson & Kearley, 2013 ▸). Our results (Fig. 14 ▸) exhibit no obvious *Q*-dependence at 275 or 300 K, which begs the question of whether we have really observed melting or simply amorphization, although the former is more plausible.

The answer to this may lie in recent observations that aqueous solutions of Mg^2+^ and SO_4_^2−^, in particular, more so than any other cation–anion pair, have a disproportionately large effect on the dynamics of water extending beyond the first hydration shell of the ions (Tielrooij *et al.*, 2010 ▸; Verde *et al.*, 2016 ▸). Such solutions, even when quite dilute, are highly structured due to the formation of solvent–ion pairs and solvent-separated ion pairs with cooperatively locked hydrogen-bonded water networks; these act to slow reorientational motion and inhibit diffusion. Indeed the lifetimes of solvent-separated ion pairs in MgSO_4_(aq) were found in molecular-dynamics simulations to be in the range 25–50 ps (Verde *et al.*, 2016 ▸), depending on concentration, values that are similar to the time-scales indicated by our observed QENS broadening.

It would be useful to carry out an ambient-pressure QENS study on aqueous MgSO_4_ solutions to determine whether this signature can be reproduced.

**Figure 15:**
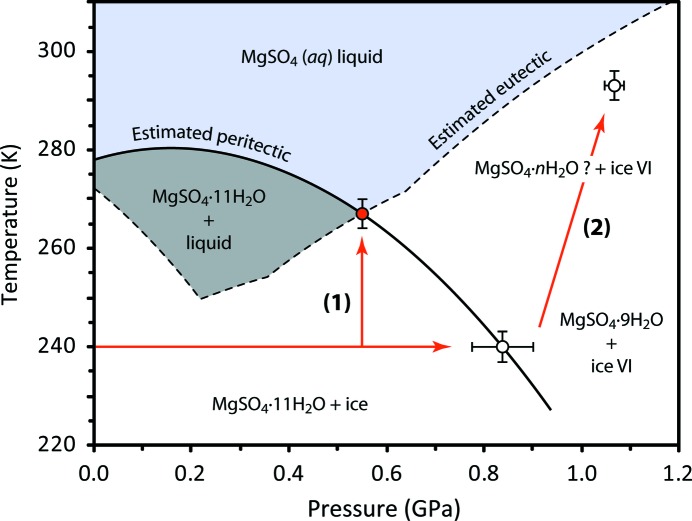
High-pressure phase diagram of MgSO_4_·11D_2_O as deduced from this work. The solid red lines depict the *P*,*T* paths followed in our three experiments (note that the two higher-pressure experiments followed the same path); the open circles report transitions seen in the Paris–Edinburgh press and the filled circle the transition seen in the gas cell. The location of the binary MgSO_4_–D_2_O system eutectic is estimated by assuming a uniform melting point depression with respect to pure D_2_O ice (*cf.* Bridgman, 1935 ▸; Pistorius, 1968 ▸). The solid curve represents our proposed peritectic, which passes through the known room-pressure incongruent melting point and the two high-pressure transitions. The label (1) indicates the sequence of diffraction patterns given in Fig. 12 ▸. The label (2) indicates the sequence of diffraction patterns given in Fig. 9 ▸. The ‘error’ bars on the points reflect the size of the *P*–*T* increments between successive diffraction patterns (the *P* and *T* loci of which have their own, smaller, uncertainties) and are therefore simply ‘bracketing’ the phase transition pressures and temperatures.

The apparently simultaneous melting of both the meridianiite and ice components of the specimen to liquid + unknown hydrate implies that the transition marks the intersection of meridianiite’s high-pressure peritectic with the system’s high-pressure eutectic (Fig. 15 ▸). Indeed this agrees with the estimated location of the eutectic (dashed line), based on a uniform depression of the water-ice melting point with pressure. Combined with the known room-pressure peritectic and the observed dissociation point observed on PEARL/HiPr, then the envelope of meridianiite’s stability must be defined by the solid curve shown in Fig. 15 ▸. The convex upward nature of this phase boundary is remarkably similar to the incongruent pressure-melting curve of mirabilite (see Fortes, Brand *et al.*, 2013 ▸, and references therein). The open circle at the end of path 2 represents the *P*,*T* locus of the diffraction pattern uppermost on Fig. 9 ▸ where it appears that ice in the sample is beginning to melt.

One of the most salient aspects of Fig. 15 ▸ is that the region of stability of meridianiite + brine in the MgSO_4_–H_2_O binary phase diagram increases, from ∼ 5 K at ambient pressure to ∼ 30 K at 200 MPa. Indeed the region from 100 to 400 MPa is representative of conditions in subsurface brine oceans in large icy satellites; the wide stability field of meridianiite under these circumstances supports the hypothesis that this will be an abundant rock-forming mineral produced as concentrated oceans cool and freeze over geological time.

Our interpretation differs from that of Hogenboom (1995 ▸) and Dougherty *et al.* (2007 ▸), particularly in relation to the meridianiite peritectic. Those authors reported a well determined eutectic for the MgSO_4_–H_2_O system up to 400 MPa, which agrees well with our estimated eutectic by virtue of being roughly parallel to the high-pressure ice-melting curve. The magnitude of the freezing-point depression was found to increase slightly with pressure and they observed only a metastable extension of the ice III melting curve in lieu of ice V melting at the highest pressures. The main difference with our work relates to the peritectic, which Hogenboom (1995 ▸) admit was very difficult to measure with confidence. The few points they report show that the peritectic follows the high-pressure melting curves of ice Ih and ice III, *maintaining* the very narrow stability field of meridianiite. However, Hogenboom (1995 ▸) also claimed to identify a new hydrate, having what they infer to be a higher hydration state than 11 H_2_O, on the liquidus at high pressure. Specifically, they placed the melting point of this hydrate at 248.5 MPa at 282 K, which is within a few degrees of our estimated meridianiite peritectic at the same pressure. Since Hogenboom (1995 ▸) could only identify phases optically and their density values, as we demonstrated earlier, are of dubious accuracy (whereas we observe the crystal structure unambiguously), it seems more plausible that their ‘new’ hydrate is actually meridianiite, which would bring their work into agreement with ours.

## 4. Discussion and concluding remarks   

To summarize, we have carried out the first experimental study, using medium- and high-resolution neutron powder diffraction, to determine the elastic properties of magnesium sulfate undecahydrate and its phase behaviour as a function of pressure. The derived elastic properties compare satisfactorily with those predicted by an earlier computational study using DFT. Whilst we have been able to draw some general conclusions as to the mechanism by which the crystal takes up strain under hydrostatic stress, the DFT calculations reported by Brand (2009 ▸) provide insight that is difficult, if not impossible, to determine experimentally into the pressure-induced breakdown of meridianiite. *In silico*, the trend seen experimentally in the shape and orientation of the strain figure continues; over the calculated pressure range from 2.5 to 5 GPa, however, the stiffest direction in the crystal (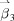) adopts a negative linear compressibility, whilst the other two directions become substantially more compressible and the strain figure continues its ‘nose up’ pitch until θ is close to 90°. The consequence of this is a dramatic decrease in the overall stiffness of the material, the bulk modulus dropping to just 15 GPa at a calculated pressure of 5 GPa. Above 5 GPa, the crystal becomes much stiffer (*K* = 55 GPa) and more elastically isotropic than in the region below 5 GPa. The most surprising aspect of the structural reorganization associated with these enormous changes in elastic properties is how small it is: there are some small rotations of the coordination polyhedra up to 2.5 GPa, which drive a shift in the acceptor atom of one hydrogen bond. The atom labelled O*w*8 (see Fortes *et al.*, 2008 ▸, for a labelled illustration of the structure) donates a hydrogen bond to one of the sulfate O atoms, O4, below 2.5 GPa, but strains to breaking point and then reforms at 5 GPa to another nearer (by virtue of ongoing polyhedral rotation) water oxygen, O*w*3, which is coordinated to an Mg^2+^ ion. This very minor change in hydrogen-bond acceptor does have one important effect, which explains the resulting change in elasticity, and that is to connect together the tips of adjacent water hexadecamers, roughly speaking along the *c*-axis, into an infinite oligomeric chain.

The onset of the structural changes in the simulations is marked by the compressibility of 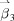 going to zero (and then negative). Extrapolation of the experimental 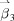 shows that this direction will transition to negative compressibility at 1.1 – 1.2 GPa, which is close to the observed dissociation around 0.8 – 0.9 GPa. Hence the origin of the dissociation may be an elastic instability in the structure brought about by an impetus on behalf of the hydrogen-bond network to extend itself, which is best resolved by expelling two interstitial water molecules from the structure.

Our observations of the high-pressure phase behaviour allow us to sketch out a phase diagram that we believe reconciles some observations in the literature and exhibits similarities to the well determined phase behaviour of related substances. It would still be desirable to probe the purported wide stability field of meridianiite in the 100 – 500 MPa region and to measure the density of the solid and Δ*V*_melting_ along our proposed peritectic. Outstanding questions concerning the identity of some new phases observed at high pressure remain, although we have shown that at least one of the high-pressure products is MgSO_4_·9H_2_O. Whether this enneahydrate is stable or metastable (it is certainly metastable at room pressure) is yet to be determined.

## 5. Related literature   

References cited in the supporting information include: Bridgman (1923 ▸, 1945*a* ▸,*b* ▸), D’Heurle *et al.* (1963 ▸), van Duijn & van Galen (1957 ▸), Feder & Nowick (1958 ▸), Goens & Weerts (1936 ▸), Kuznetsov *et al.* (2002 ▸), Mao & Bell (1978 ▸), Mao *et al.* (1990 ▸), Miller & Schuele (1969 ▸), Nix & MacNair (1942 ▸), Pautomo (1963 ▸), Prasad & Wooster (1956 ▸), Rubin *et al.* (1962 ▸), Stokes & Wilson (1941 ▸), Swift & Tyndall (1942 ▸), Touloukian *et al.* (1975 ▸), Uffelman (1930 ▸), Vaida & Kennedy (1970 ▸), Vold *et al.* (1977 ▸), Waldorf & Alers (1962 ▸).

## Supplementary Material

Supporting tables and explanatory information on Pb EoS. DOI: 10.1107/S2052520616018254/fx5002sup1.pdf

## Figures and Tables

**Table 1:** Parameters obtained by least-squares fitting (using *OriginPro*), with instrumental weighting, either a quadratic polynomial expression of the form *X* + *YP* + *ZP* ^2^ (*a*-axis, *b*-axis) or a linear expression of the form *X* + *YP* (*c*-axis, α, β, γ) to the unit-cell parameters listed in Table S1

	*X* (Å, °)	*Y* (Å, ° MPa^−1^)	*Z* (Å MPa^−2^)	Adjusted *R* ^2^
*a*-axis	6.7503 (6)	−6.2 (5) × 10^−6^	1.6 (8) × 10^−10^	0.9884
*b*-axis	6.8117 (2)	−1.72 (2) × 10^−5^	1.6 (4) × 10^−10^	0.9998
*c*-axis	17.2912 (5)	−1.33 (2) × 10^−5^	–	0.9973
α	88.136 (3)	1.05 (1) × 10^−4^	–	0.9987
β	89.494 (3)	1.7 (1) × 10^−5^	–	0.9512
γ	62.678 (4)	−5.6 (1) × 10^−5^	–	0.9910

**Table 2:** Derived linear and volumetric compressibilities (*B*) and incompressibilities (*K*) found by fitting linear expressions to the pressure dependences of the compressibility tensor’s eigenvalues

	*B*_0_ (GPa^−1^)	*B*′	*K*_0_ (GPa)	*K*′
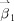	9.6 × 10^−3^	−4.4 × 10^−3^	104	49
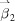	3.35 × 10^−2^	−3.2 × 10^−3^	30	3
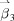	3.4 × 10^−3^	−5.4 × 10^−4^	293	47
β_V_	4.66 × 10^−2^	−8.1 × 10^−3^	21.5	3.8
