# Supplementary material for: Isothermal equation of state and high-pressure phase transitions of synthetic meridianiite (MgSO4·11D2O) determined by neutron powder diffraction and quasielastic neutron spectroscopy
Source: Acta Crystallogr B Struct Sci Cryst Eng Mater. 2017 Jan 31;73(Pt 1):33–46. doi: 10.1107/S2052520616018254 (PMC5289305; doi:10.1107/S2052520616018254)
Supplement: Supplementary file 1 [file b-73-00033-sup1.pdf]

STRUCTURAL SCIENCE
CRYSTAL ENGINEERING
MATERIALS

Volume 73 (2017)

Supporting information for article:

Isothermal equation of state and high-pressure phase transitions of synthetic meridianiite (MgSO4·11D2O) determined by neutron powder diffraction and quasielastic neutron spectroscopy

Andrew Fortes, Felix Fernandez-Alonso, Matthew Tucker and Ian Wood

Table S1

Refined unit-cell parameters of $MgSO_4 \cdot 11D_2O$ as a function of pressure.

T (K)	P (MPa)	*a* (Å)	*b* (Å)	*c* (Å)	α (°)	β (°)	γ (°)	V (Å^3)
**Fortes *et al*., (2008)**								
Uncertainty in T = ± 0.05 K, in P = n/a (ambient).								
240	0.1	6.7495(1)	6.8115(1)	17.2920(3)	88.135(1)	89.491(1)	62.687(1)	705.97(1)
**Experiment 1: PEARL/HiPr, 2008**								
Uncertainty in T = ± 1 K, in P ≈ 5 %								
240	192	6.735(1)	6.776(1)	17.269(3)	88.38(2)	89.60(2)	62.60(2)	699.4(3)
240	248	6.735(1)	6.769(1)	17.260(3)	88.40(1)	89.59(2)	62.57(1)	698.1(3)
240	257	6.732(2)	6.764(2)	17.255(4)	88.42(2)	89.56(2)	62.57(2)	697.1(3)
240	260	6.733(2)	6.760(2)	17.251(4)	88.47(2)	89.60(2)	62.53(2)	696.5(3)
240	496	6.721(1)	6.728(1)	17.225(2)	88.70(1)	89.60(1)	62.39(1)	690.0(1)
240	773	6.710(1)	6.691(1)	17.191(3)	88.97(1)	89.63(2)	62.23(2)	682.8(2)
**Experiment 2: PEARL/HiPr, 2009**								
Uncertainty in T = ± 1 K, in P ≈ 5 %								
240	514	6.7234(4)	6.7250(4)	17.224(1)	88.733(5)	89.578(6)	62.339(5)	689.59(8)
**Experiment 3: OSIRIS, 2011**								
Uncertainty in T = ± 0.1 K, in P = 0.3 %.								
240	48.0	6.7479(2)	6.8036(2)	17.2850(6)	88.175(3)	89.509(3)	62.650(2)	704.48(2)
240	103.5	6.7453(2)	6.7947(2)	17.2767(5)	88.246(2)	89.524(3)	62.606(2)	702.69(2)
240	149.7	6.7430(2)	6.7867(2)	17.2680(5)	88.306(2)	89.519(3)	62.584(2)	701.16(2)
240	200.2	6.7390(2)	6.7784(2)	17.2647(5)	88.340(2)	89.530(3)	62.550(2)	699.55(2)
240	252.0	6.7366(2)	6.7694(2)	17.2554(5)	88.397(2)	89.532(3)	62.523(2)	697.84(2)
240	305.5	6.7325(2)	6.7603(2)	17.2515(5)	88.460(2)	89.556(2)	62.497(2)	696.18(2)
240	351.0	6.7292(2)	6.7533(2)	17.2450(5)	88.502(2)	89.561(2)	62.468(2)	694.69(2)
240	409.8	6.7252(2)	6.7430(2)	17.2399(5)	88.569(2)	89.566(3)	62.461(2)	692.99(2)
240	451.8	6.7254(2)	6.7367(2)	17.2311(5)	88.607(2)	89.561(3)	62.439(2)	691.88(2)
240	499.8	6.7236(2)	6.7295(2)	17.2242(5)	88.661(2)	89.566(3)	62.404(2)	690.48(2)
240	550.0	6.7218(1)	6.7218(1)	17.2184(3)	88.709(1)	89.582(2)	62.371(1)	689.08(1)

Pressure calibration using a revised equation of state for Pb

The Pb equation of state employed in this work was derived from a synthesis of literature values for the ambient-pressure thermal expansivity, and ultrasonic determinations of the temperature dependence of the bulk modulus and its first pressure derivative (see overleaf). The principal improvement in our implementation of this equation of state over that described previously (Fortes *et al.*, 2007, 2012) lies in the robust propagation of errors.

The pressure is determined using a 3rd-order Birch-Murnaghan equation of state,

$$P_{V,T} = \frac{3}{2}K_{0,T}\left(x^{7/3} - x^{5/3}\right)\cdot\left[1 + \frac{3}{4}\left(K'_{0,T} - 4\right)\left(x^{2/3} - 1\right)\right]$$

where $x = V_{0,T} / V_{P,T}$; and the unit-cell volume, $V_{0,T}$, the isothermal bulk modulus, $K_{0,T}$, and its first pressure derivative, $K'_{0,T}$, are found from temperature dependent polynomials; The second pressure derivative of the bulk modulus, $K''_{0,0}$, is assumed to be independent of temperature.

$V_{0,T} = V_{0,0} + aT + bT^2$	$V_{0,0}$	121.41813 ± 0.00013 Å^3
	a	$1.05822 \pm 0.00007 \times 10^{-2}$ Å^3 K^{-1}
	b	$3.493 \pm 0.004 \times 10^{-6}$ Å^3 K^{-2}
$K_{0,T} = K_{0,0} + cT + dT^2$	$K_{0,0}$	41.725 ± 0.009 GPa
	c	$-2.544 \pm 0.004 \times 10^{-2}$ GPa K^{-1}
	d	$-2.76 \pm 0.24 \times 10^{-6}$ GPa K^{-2}
$K'_{0,T} = K'_{0,0} + eT$	$K'_{0,0}$	5.39 ± 0.25
	e	0.0011 ± 0.001 K^{-1}

In a typical high-pressure experiment carried out on PEARL using the Paris-Edinburgh press, it is usual to obtain an uncertainty of ~ 0.05 % in the Pb unit-cell volume and ~ 0.3 % in temperature; the propagated error in the pressure is then approximately 3 %.

References

Our previous application of the Pb equation of state is given in:

Fortes, A. D., I. G. Wood, M. Alfredsson, L. Vočadlo, K. S. Knight, W. G. Marshall, M. G. Tucker, and F. Fernandez-Alonso (2007) The high-pressure phase diagram of ammonia dihydrate. *High Press. Res.* **27**(2), 201-212 (doi:10.1080/08957950701265029), and Corrigendum (doi:10.1080/08957959.2012.673603).

Thermal expansion of Pb

D'Heurle, R. Feder, and A. S. Nowick (1963) Equilibrium concentration of lattice vacancies in lead and lead alloys. *J. Phys. Soc. Japan Suppl. II.*, **18**, 184-190.

Feder, R., and A. S. Nowick (1958) Use of thermal expansion measurements to detect lattice vacancies near the melting point of pure lead and aluminium. *Phys. Rev*. **109**(6), 1959-1963 (doi:10.1103/PhysRev.109.1959).

Nix, F. C., and D. MacNair (1942) The thermal expansion of pure metals. II: molybdenum, palladium, silver, tantalum, tungsten, platinum, and lead. *Phys. Rev*. **61**(1-2), 74-78 (doi:10.1103/PhysRev.61.74).

Rubin, T., H. L. Johnston, and H. W. Altman (1962) The thermal expansion of lead. *J. Phys. Chem.* **66**(2), 266-268 (doi:10.1021/j100808a018).

Stokes, A. R., and A. J. C. Wilson (1941) The thermal expansion of lead from 0 °C to 320 °C. *Proc. Phys. Soc.* **53**(6), 658-662 (doi:10.1088/0959-5309/53/6/304).

Touloukian, Y. S., Kirby, R. K., Taylor, R. E., & Desai, P. D. (1975) In, *Thermal Expansion, Metallic Elements and Alloys, Vol. 12 of TPRC series on thermophysical properties of matter* (Eds. S. Touloukian and C. Y. Ho), Plenum, New York.

Uffelman, F. L. (1930) The expansion of metals at high temperatures. *Phil Mag.* [Ser. 7], **10**(65), 633-659 (doi:10.1080/14786443009461615).

Van Duijn, J., and J. Van Galen (1957) Influence of vacancies on the thermal expansion of lead near the melting point. *Physica* **23**(6-10), 622-624 (doi:10.1016/S0031-8914(57)93541-3).

Elastic constants of Pb

Goens, E., and J. Weerts (1936) Hauptelastizitätskonstantan des Einkristalls von Kupfer, Gold und Blei. *Physikalische Zeitschrift* **37**, 321-326.

Miller, R. A., & Schuele, D. E. (1969) The pressure derivatives of the elastic constants of lead. *J. Phys. Chem. Solids* **30**, 589-600 (1969) (doi:10.1016/0022-3697(69)90014-6).

Pautomo, Y. (1963) On the temperature variation of volume. *Ann. Acad. Sci. Fennicæ Ser. A VI, Phys*. **129**, 7-45.

Prasad, S. C., and W. A. Wooster (1956) The elasticity of single crystals of lead. *Acta Cryst.* **9**(1), 38-42 (doi:10.1107/S0365110X56000073).

Swift, I. H., and E. P. T. Tyndall (1942) Elasticity and creep of Pb single crystals. *Phys. Rev.* **61**(5-6), 359-364 (doi:10.1103/PhysRev.61.359).

Vold, C. L., M. E. Glicksman, E. W. Kammer, and L. C. Cardinal (1977) The elastic constants for single-crystal lead and indium from room temperature to the melting point. *J. Phys. Chem. Solids* **38**(2), 157-160 (doi:10.1016/0022-3697(77)90159-7).

Waldorf, D. L., & Alers, G. A. (1962). Low temperature elastic moduli of lead. *J. Appl. Phys*. **33**(11), 3266-3269 (1962) (doi:10.1063/1.1931149).

High-pressure studies of Pb

Bridgman, P.W. (1923) The compressibility of thirty metals as a function of pressure and temperature. *Proc. Am. Acad. Arts Sci*. **58**(5), 165-242 (doi:10.2307/20025987).

Bridgman, P.W. (1945a) The compression of twenty-one halogen compounds and eleven other simple substances to 100,000 kg/cm^2. *Proc. Am. Acad. Arts Sci*. **76**(1), 1-7 (doi:10.2307/20023492).

Bridgman, P.W. (1945b) The compression of sixty-one solid substances to 25,000 kg/cm^2, determined by a new rapid method. *Proc. Am. Acad. Arts Sci.* **76**(1), 9-24.

Mao, H. K., and P. M. Bell (1978) Study of lead at high pressure: compressibility and fixed-point transition between the FCC and HCP polymorphs under varying degrees of non-hydrostatic stress. *Carnegie Inst. Yearbook* **77**, 842-848 (http://archive.org/details/yearbookcarne77197778carn).

Mao, H. K., Y. Wu, J. F. Shu, R. J. Hemley, and D. E. Cox (1990) High-pressure phase transitions and equation of state of lead to 238 GPa. *Solid State Comm.* **74**(9), 1027-1029 (doi:10.1016/0038-1098(90)90479-U).

Vaida, S. N., and G. C. Kennedy (1970) Compressibility of 18 metals to 45 kbar. *J. Phys. Chem. Solids* **31**(10), 2329-2345 (doi:10.1016/0022-3697(70)90247-7).

Kuznetsov, A. Z., Dmitriev, V., Dubrovinsky, L., Prakapenka, V., Weber. H. –P. (2002) FCC-HCP boundary in lead. *Solid State Comm*. **122**(3-4), 125-127 (doi:10.1016/S0038-1098(02)00112-6).
